# Supplementary material for: Cost savings associated with extended battery longevity in cardiac resynchronization therapy defibrillators
Source: Heart Rhythm O2. 2024 Sep 20;5(11):755–61. doi: 10.1016/j.hroo.2024.09.008 (PMC11624373; doi:10.1016/j.hroo.2024.09.008)
Supplement: Supplemental Material [file mmc1.docx]

**Supplemental Material:**

**Appendix 1**

List of Complications Used by Schmier et al. (2017)

| Category | Description |
| --- | --- |
| Complications, infection | Septicemia  Endocarditis  Cellulitis  Fever  Shock  Bacteremia  Infection due to device |
| Complications, non-infection | Chest tube insertion  Pericardiocentesis  Pulmonary embolism  Hemopericardium  Cardiac tamponade  Unspecified disease of pericardium  Pleural effusion  Pneumothorax  Respiratory arrest  Hematoma |

Calculation of Costs to Medicare for Implantation and Replacement:

Inpatient Initial Implantation:

| DRG Code | Claim Volume (Number), 2019-2021 | Claim Volume (Percent) | Medicare 2023 Reimbursement Amount (USD) | Weighted Average |
| --- | --- | --- | --- | --- |
| 222 | 1,533 | 14% | $52,520 | $42,485 |
| 223 | 313 | 3% | $35,798 |  |
| 224 | 1,409 | 13% | $48,628 |  |
| 225 | 708 | 7% | $34,693 |  |
| 226 | 3,900 | 37% | $43,907 |  |
| 227 | 2,731 | 26% | $34,439 |  |

DRG, diagnosis-related group; USD, US dollar

Outpatient Initial Implantation:

| APC Code | Medicare 2023 Reimbursement Amount (USD) |
| --- | --- |
| 5232 | $32,076 |

APC, ambulatory payment classification

Weighted Average of Inpatient and Outpatient Initial Implantation Costs

| Site of Service | Claim Volume (Number), 2019-2021 | Claim Volume (Percent) | Average Medicare 2023 Reimbursement (USD) | Weighted Average |
| --- | --- | --- | --- | --- |
| Inpatient | 10,594 | 22.7% | $42,485 | $34,436 |
| Outpatient | 36,136 | 77.3% | $32,076 |  |

Data Source: Centers for Medicare and Medicaid Services. 2019-2021 Medicare 100% standard analytic files. DRG/APC Code list.

Weighted Average of Inpatient and Outpatient Replacement Costs

| Site of Service | Claim Volume (Number), 2019-2021 | Claim Volume (Percent) | Average Medicare 2023 Reimbursement (USD) | Weighted Average |
| --- | --- | --- | --- | --- |
| Inpatient (DRG 245) | 1,127 | 3.4% | $33,447 | $32,123 |
| Outpatient (APC 5232) | 31,842 | 96.6% | $32,076 |  |

Weighted Average of Follow-up Visit Costs

| Follow-Up Visit CPT Code | Claim Volume (Number), 2019-2021 | Claim Volume (Percent) | Average Medicare 2023 Reimbursement for Facility Location (USD) | Weighted Average |
| --- | --- | --- | --- | --- |
| 93289 | 2,116 | 6.9% | $73.87 | $82.30 |
| 93284 | 4,189 | 13.6% | $107.76 |  |
| 99212 | 2,174 | 7.1% | $35.58 |  |
| 99213 | 11,416 | 37.1% | $66.08 |  |
| 99214 | 10,214 | 33.2% | $97.60 |  |
| 99215 | 697 | 2.3% | $143.34 |  |

Data Source: Centers for Medicare and Medicaid Services. 2019-2021 Medicare 100% standard analytic files. DRG/APC Code list.

**Appendix 2: Sensitivity Analysis Input Parameters**

| Parameter | 2.1Ah | 1.0Ah | 1.6Ah | Notes |
| --- | --- | --- | --- | --- |
| Patient survival (Scenario 2)  Year 0  Year 1  Year 2  Year 3  Year 4  Year 5  Year 6 | 100%  88%  79%  71%  62%  54%  45% | 100%  88%  79%  71%  62%  54%  45% | 100%  88%  79%  71%  62%  54%  45% | Source: Saxon et al. (2010); Author’s assumption for Year 6: based on the trend that every year the survival probability drops by 8% to 9%; it is assumed the survival probability drops by 9% from Year 5 to Year 6. |
| Patient survival (Scenario 3)  Year 0  Year 1  Year 2  Year 3  Year 4  Year 5  Year 6  Year 7  Year 8  Year 9  Year 10  Year 11  Year 12  Year 13  Year 14  Year 15 | 100%  95%  90%  85%  81%  77%  72%  67%  62%  57%  52%  47%  42%  37%  32%  27% | 100%  95%  90%  85%  81%  77%  72%  67%  62%  57%  52%  47%  42%  37%  32%  27% | 100%  95%  90%  85%  81%  77%  72%  67%  62%  57%  52%  47%  42%  37%  32%  27% | Source: Yao et al. (2007); Author’s assumption (extrapolation) for Year 7-15. |
| Event-free battery survival  Year 0  Year 1  Year 2  Year 3  Year 4  Year 5  Year 6  Year 7  Year 8  Year 9  Year 10-15 | 100%  100%  98%  98%  95%  90%  77%  45%  26%  10%  0% | 100%  100%  99%  92%  74%  36%  10%  3%  0%  0%  0% | 100%  100%  100%  100%  90%  69%  44%  0%  0%  0%  0% | Source: Alam et al. (2017) through Year 6; author’s expertise for Years 7 through 15. |
| Incidence of complication  Complication first year after primary implant  Complication first year after replacement  Infection first year after primary implant  Infection first year after replacement | 7%  3%  4%  7% | 7%  3%  4%  7% | 7%  3%  4%  7% | Source: Schmier et al. (2017) upper bound |
| Complication cost to Medicare  Complication  Infection | $1,427  $50,671 | $1,427  $50,671 | $1,427  $50,671 | Source: Schmier et al. (2017) upper bound; 2023 USD |
| Medicare costs of CRT-D  Initial implantation  Replacement  Follow-up visit (per visit; 4 visits per year) | $34,436 +/- 20%  $32,123 +/- 20%  $82 +/- 20% | | | Sources:  Costs = base case +/- 20%  Number of visits = no change from base case |

CRT-D, cardiac resynchronization therapy defibrillator; USD, US dollars

**Appendix 3. Sensitivity Analysis Results**

|  | Average Per-patient Medicare Costs over 6-year Follow-up | | |  |  |
| --- | --- | --- | --- | --- | --- |
| Scenario | 2.1Ah | 1.0Ah | 1.6Ah | Difference between 1.0Ah and 2.1Ah, USD (%) | Difference between 1.6Ah and 2.1Ah, USD (%) |
| Scenario 1: Base Case | $41,527 | $56,647 | $48,515 | $15,120 (36%) | $6,988 (17%) |
| Scenario 2: Patient survival (Saxon) | $39,757 | $50,572 | $44,483 | $10,815 (27%) | $4,727 (12%) |
| Scenario 3: Patient survival (Yao) +15 years of follow-up | $60,994 | $75,266 | $67,491 | $14,272 (23%) | $6,947 (11%) |
| Scenario 4a: Procedure costs -20% | $34,640 | $49,760 | $41,627 | $15,120 (44%) | $6,988 (20%) |
| Scenario 4b: Procedure costs +20% | $48,414 | $63,534 | $55,402 | $15,120 (31%) | $6,988 (14%) |
| Scenario 5a: Replacement costs -20% | $40,555 | $52,742 | $46,188 | $12,187 (30%) | $5,632 (14%) |
| Scenario 5b: Replacement costs +20% | $42,498 | $60,551 | $50,842 | $18,054 (42%) | $8,344 (20%) |
| Scenario 6: Complication rate (Schmier upper bound) | $42,331 | $57,996 | $49,571 | $15,665 (37%) | $7,240 (17%) |
| Scenario 7: Complication costs (Schmier upper bound) | $42,058 | $57,471 | $49,181 | $15,412 (37%) | $7,123 (17%) |

All values are in 2023
